# Dentifrices*Read before the National Dental Association, Boston, Massachusetts, August 23-27, 1920. Reprinted in part from *Journal N. D. A.*, July, 1921.

**Published:** 1921-10

**Authors:** C. Edmund Kells


### DENTIFRICES*

BY C. EDMUND KELLS, D.D.S.

I believe it can be safely stated that it is now realized that one of the greatest factors, if not the greatest single factor, in the general health of a person from infancy through to old age is the condition of the mouth or the teeth. Barring unusual cases, the condition of the teeth usually depends in a great measure upon the care taken

---

* Read before the National Dental Association, Boston, Massachusetts, August 23-27, 1920. Reprinted in part from *Journal N. D. A.*, July, 1921.

of them, consequently we arrive at the conclusion that the care of the teeth from the moment of their eruption is of paramount importance. Therefore the question of dentifrices should be considered one of the most vital questions of the day.

THE DENTIFRICE OF TODAY

There are undoubtedly some dentifrices upon the market that were originally prepared in good faith, because there was a necessity for them; but, undoubtedly, most of the recent products in this direction were originated solely for the purpose of separating an unwary public from its money.

The history of most of these products undoubtedly is about as follows: The idea is first conceived by some "Mr. Wallingford" that millions of people brush their teeth, and these millions will use some dentifrice. Then why not get into the game, originate some dentifrice, advertise it, and sell it to them? Nothing is easier. The next idea is to endeavor to have some novel and catchy feature—a *feature* mind you, not necessarily a fact—about the dentifrice for which the public "will fall." Once the manufacture is begun, the dental profession is sampled, and the education of the public is commenced by startling advertisements in the papers.

What of the profession? I believe I can safely say that 95 per cent of the dental profession has not the slightest idea as to what a dentifrice should or should not contain; that probably this 95 per cent adopt first one and then another of these dentifrices as fast as they are placed upon the market, irrespective of their composition, and as they are induced to do so by their silver-tongued representatives.

Let each one ask himself the question, "What do I really know of the dentifrice that I am using and that I recommend to my patients?" and see what the answer will be. As a matter of fact, why should any one of us really know anything about dentifrices? Who has educated us upon the subject? What are our colleges doing for prophylaxis and dentifrices? Practically nothing I should say. Or if they are doing something for the subject of dentifrices, it is so smothered up and kept so dark that probably even the students do not appreciate their efforts.

I have before me the bulletin for 1920-21 of one of the leading colleges of the country—probably no other school outranks it. I find that it lists forty-five professors, lecturers, and instructors, eight assistants, and one examiner. These fifty-four men are scheduled to lecture upon all the regular topics usually taught in a dental college, including English, drawing, jurisprudence, and ethics. The examination of patients requires the services of a D.D.S., while an M.D. is a special research investigator. All this, and never a mention of prophylaxis or dentifrices. All this array of talent for everything except prophylaxis—prophylaxis, the very keystone upon which the arch of modern dentistry should depend, is completely ignored. Yet it is not possible for the dental graduate to leave that college with a proper appreciation of dentifrices and their value. It may be possible that under the topic of oral hygiene the colleges do pay more attention to the question of dentifrices than I have given them credit for.

If any one other than Dr. Pickerill has advanced any ideas upon the subject, who is he? Is he not some one who has placed some dentifrice of his own upon the market. And upon what does he base his knowledge of the subject? Now and then we find a dentist who imagines that there is a necessity for another dentifrice, or who possibly would like additional revenue; he therefore concocts a formula of his own, manufactures it, and furnishes it to his patients and to the public. Right or wrong, that man is to be commended. He at least has some ideas of his own, even if they are wrong.

Not much was heard upon this question of dentifrices until 1912, when Dr. Pickerill[1] published a brilliant work

---

[1] H. P. Pickerill, M.D., Ch.B., M.D.S., (Birm.) L.D.S. (Eng.). *The Prevention of Dental Caries and Oral Sepsis.*

and his theories were accepted by some apparently without giving them serious consideration. When this work on *Prevention of Dental Caries and Oral Sepsis* made its appearance, it gave to many men a new trend of thought. Pickerill's ideas, from their very nature, must have been purely theoretical. In fact, he admits in his very first chapter that his conclusions were drawn a priori, and a priori conclusions should be impossible for a research worker. I say his ideas must have been purely theoretical, because at the time his book was published he was still a young man, had been in practice but a few years, and thus could not possibly have had any clinical experience, especially as he was not located in one place for even a short time. Thus it was impossible for him to have followed up his patients in order to note the results of his practice. However we may disagree with him upon all his main points, we must admit that he has given the profession a most interesting work, and those who have not read it have missed a great deal. To quote from this work: ''We are therefore driven to the inevitable conclusion that alkaline dentifrices and mouth washes for the prevention of dental caries must be abandoned, and we further conclude that some substance which is a salivary stimulant should be used in order to promote and educate the activity of the salivary glands. Now it has also been conclusively proven that the best substance for this purpose is acids and there is not the slightest reason why the best should not be used in this case.''

Upon reading such a dogmatic statement, the question at once arises, ''Who is Pickerill?'' The answer has already been given—a wonderfully brilliant and earnest worker, but a young man of theories, without any practical clinical experience. Evidently Dr. Pickerill's attention was drawn to two facts; the one, that dental caries were just as prevalent at that time, if not more so than they were one hundred years before; and second, that alkaline dentifrices had always been used to combat the disease.

Both of these statements are undoubtedly true, but he then jumps to the conclusion that the "treatment was radically wrong," and therein lies his mistake. Dental caries are prevalent today, not because of the use of alkaline dentifrices, but rather in spite of them. As a matter of fact, caries of the teeth have increased among countless thousands who never used any dentifrice whatever. Dental caries may have increased among another group of countless thousands who used alkaline dentifrices, but how did they use them? What proportion of the more educated classes, who even pretend to take care of their teeth, do so with any degree of thoroughness? The mere fact that the normal condition of the mouth is either neutral or slightly alkaline and that the introduction of an acid into the mouth induces an increased flow of an alkaline saliva is proof positive that the acid is harmful. Nature, when normal, is continuously on guard, and whenever anything harmful gains entrance into the body, she at once endeavors to fight it off.

Pickerill advocates the idea that a dentifrice should be a salivary stimulant. With all due deference to him and his following, I do not hesitate to affirm that a salivary stimulant is the very last ingredient a dentifrice should contain. Where is the dentist who cannot clean and polish the teeth in the upper arch of any patient when he can keep this operative field comparatively dry and free from saliva, better than he can the lower teeth, which in some patients are practically flooded with saliva all the time? The slimy nature of the saliva renders it most difficult, or even impossible, to polish the teeth. Such being the case when the dentist is trying to clean the teeth of a patient, why do not the same conditions obtain when the patient is trying to clean his teeth himself? They certainly do.

Any experienced dentist will indeed insist that the less saliva there is in the mouth during the process of brushing the teeth the better for the patient, and that instead of a dentifrice containing an acid fruit juice or any other stimulating substance in order to increase the flow of saliva, it should, if possible, contain some ingredients to produce just the reverse effect, to reduce the flow of saliva. This acid theory of Pickerill's was the one great idea that has inspired some of our more recent creations in the way of dentifrices, and their sponsors would endow these acid fruit juices with superhuman intelligence, a trait that Dr. Pickerill did not discover. We all know by our own experience that acid fruit juices destroy the teeth and we need no postgraduate course upon the subject. It is no unusual occurrence for a patient to state that the teeth are all "on edge" and exceedingly sensitive. The first inquiry is as to diet, and we invariably find that acid fruits, oranges, grape fruit, and lemons, have been recently used in unusually large quantities. A change of diet is recommended, and also alkaline mouth washes, and in a week or two the mouth is again in normal condition.

How many of us but have seen the literal *wrecks* that schoolgirls have made of their teeth by eating lemons and sugar or salt. But when these fruit acids are incorporated into these dentifrices they become endowed with superhuman power, because the manufacturer tells us—prints it in fact—that all these acids do is to eat away the plaque, and the instant the plaque is dissolved—not a fraction of a second sooner nor a fraction of a second later, but just at that instant— and the acid is ready for business upon the enamel, it stops and awaits the arrival of the alkaline saliva, the flow of which it has stimulated, to neutralize it; thus the surface of the tooth is saved. Here we have an enamel-destroying agent eating its way down to the enamel and then ceasing work. One would think such a statement too absurd for belief, and yet this theory has gained a foothold where it greatly surprises us.

Professor Gies, of Columbia University, New York, unfortunately has taken up this idea of the educated acid fruits; he is advocating their use, which is much to be regretted because his opinion should carry some weight, but let us not forget that Professor Gies[2] is not now, and never was, a dentist. Let us view this, not from a theoretical standpoint, but from a common-sense point of view, that is, from that of the practical and experienced dentist. We know that acid fruit juices will dissolve the enamel. We know, and no one will deny it, that every time an acid fruit juice is taken into the mouth it will attack the enamel if in sufficient quantity and given sufficient time. Knowing these facts, we can readily see the danger of acid fruit juices used in a dentifrice two or three times a day, when the manufacturers themselves admit that they rely upon the alkaline saliva to come along at the right instant to neutralize them, and thus prevent them from doing harm. What if the neutralizing agent misses connection? This theory appears absolutely ridiculous because I believe it is reasonable to assume that many a time the acid will get a chance to attack the enamel before it becomes neutralized. Pickerill himself, however, never did make any such ridiculous claims, but believes the contrary, as the following quotation will prove: "Undoubtedly there is a short time during which the acid remains unneutralized, but this is not sufficient to cause any effect except upon the occlusal surface." This is merely a statement of opinion, and the wonder is that the acid will affect the occlusal surfaces only and not the others. As a matter of fact, I see no reason to believe that it will not affect the others as well, and from clinical evidence, I believe it does. We also find the following statement (p. 232): "There are, however, two dangers to guard against—those of using an acid either too weak or too strong. If an acid be used in too weak a solution it will do more harm than good. If an acid be used in too strong a solution it may have precisely similar effect." These admissions from the father of the acid

---

[2] William J. Gies is editor of the *Journal of Dental Research* and a member of the Biochemical Department of Columbia University, College of Physicians and Surgeons. He is author of the article on "Studies of Dentifrices," in the *Journal of Allied Dental Societies.*—EDITORIAL NOTE.

dentifrice theory would spoil the effect for me, even if I had been slightly inclined toward it.

To summarize, then, on the acid dentifrice proposition, be it powder, paste, or liquid. Dr. Pickerill says there is undoubtedly an interval of time between the use of an acid and the flow of the neutralizing alkaline saliva, during which time the acid could attack the enamel; also that the acid must be of a certain definite strength, for if weaker or stronger than this definite dilution it will do more harm than good, but he does not give us the faintest idea as to what the correct strength should be. Imagine, if you can, the thoughtful intelligent dentist using an acid in the mouth which its sponsor distinctly states must be of some unknown strength, for "if weaker or stronger" it will do more harm than good. We note the absence of these quotations from the advertisements of these acid dentifrices.

Let us write it down, then, in capital letters, that all those who use acid dentifrices—powders, paste, or liquids—do so at the risk of their teeth, we have Dr. Pickerill's written word for it, and he originated the acid dentifrice.

Let us cite an analogous case. A large room, the floor of which is covered with shavings and other combustibles, is provided with an automatic sprinkler system. A fire is started, but before it has gained any appreciable headway the sprinkler operates and the fire is extinguished. The next day another fire is started, and again it is promptly extinguished. Again the next day, and the next, and so on. Every time the fire is started, it automatically starts the flow of water and it is extinguished, but note the fact that before it is extinguished it has burned up some of the shavings. By starting the fire often enough all the shavings will be burned up, notwithstanding the fact that the sprinkler system puts out the fire each time. This exact process occurs in the mouth. Every time the harmful acid is introduced into the mouth, nature neutralizes it by an undue flow of alkaline saliva, but before that can be done it has accomplished some little harm.

### GUM-CHEWING

I thoroughly disapprove of exciting an undue flow of saliva during the brushing of the teeth; however, if for any reason an unusual flow is desired, it can best be excited by chewing gum. While I do not consider gum as a dentifrice, I believe that the chewing of gum for three minutes after meals can, in some instances, prove very beneficial. For children who bolt their food, for example, the chewing of gum for three minutes afterward not only cleanses their teeth very effectively but also gives the teeth that exercise which they would have received had their food been properly masticated. This, of course, induces a flow of saliva which might prove of benefit in assisting digestion.

### PERSONAL EXPERIENCE

I started the use of alkaline dentifrices and the use of lime water coincident with the beginning of my practice, just forty-two years ago. For forty years, I myself was a consistent user of these alkaline agents, and during the forty years I lost one perfectly good third molar and had about five fillings inserted.

During our recent critical period I received a dentifrice, free. I said to myself, "Why waste this good material, why not 'test' it out as requested?" This dentifrice containing an acid was diametrically opposed to all my practice and teachings of forty years, but appreciating the fact that it cost nothing, and that it was wicked to throw it away. I used it. At the end of the year my dentist found and filled four cavities; three of which were in the incisors which were carefully brushed and flossed every day. Forty years of alkaline dentifrices and five fillings; one year of acid plaque-digesting dentifrice and four cavities, and in a man of my age! Never again will I stray from the path blazed away forty years of satisfactory clinical results of alkaline dentifrices. Of course, this may have only been a coincidence, but why did none of the various members of my family have such a coincidence while sticking to their alkaline dentifrices? Just another case of the automatic sprinkler putting the fire out after some damage had been done!

AN IMPORTANT QUESTION

The question of dentifrices cannot be settled by scientific laboratory experiments. It must be decided upon clinical evidence. No question of greater importance to the people at large can be brought before this association than that of a suitable and safe dentifrice, and the question of the advisability of alkaline or acid preparations should not be decided by ex parte testimony, but by some thorough and exhaustive tests. I am not prepared to say just how these tests should be carried out, but it would appear to me that it would be possible to use certain institutions for that purpose. Possibly either Boston or Rochester with their magnificent Forsyth and Eastman clinics could handle such a proposition, for, undoubtedly, it is a man's-size job.

Let us suppose an institution with, say, two hundred children. Their teeth are placed in good condition and their mouths are supposedly to receive the same daily routine attention. One hundred are to use acid mouth washes five times a day, rinsing their mouths for a certain number of minutes each time, and to brush their teeth with acid dentifrices twice a day. One hundred to do the same with alkaline preparations. Carry this on for, say, two years, during which period the teeth of each child should be examined every thirty or sixty days and carefully charted. This series of experiments should be carried on in various parts of the country at the same time.

As I said before, this is a "man's-size job" and it will cost money. Are there not some men in the profession capable of handling it? Could not this association raise the necessary amount? Could it be used to any better advantage? Who will say that prevention of decay is not more desirable than reconstruction? Who will say that the prevention of decay does not lie largely in the hands of the patient? Who will say that patients should not obtain their instructions for this work from their dentists? Who is there that believes that dentists as a class have been doing their full duty to their patients in this matter?

I was taught the use of alkaline dentifrices and began my practice with the use of alkaline dentifrices and began advocated alkaline dentifrices ever since. Year by year I saw the good results obtained by their use, and I became more and more convinced that alkaline dentifrices were the dentifrices to use. As an indication of my interest in my patients' welfare, and in an endeavor to do my duty by them, I have given them printed cards of instructions for possibly thirty years or more. While the wording has been changed from time to time, the gist of the instructions has always been the same. Herewith are listed copies of these instruction cards:

### CARE OF THE TEETH

Upon rising, the mouth should be thoroughly rinsed with an alkaline mouth wash, preferably lime water, Glyco-Thymoline, or Borine.

After breakfast waxed floss silk or tape should be passed between the teeth (being careful not to snap it down hard upon the gums, which would injure them), after which the teeth should be most carefully, thoroughly and *correctly* brushed, perferably as follows:

A suitable brush should have been prescribed by the dentist. Upon this dry brush place a very small quantity of some approved *alkaline* dental cream, perferably Kolynos, S. S. White's, or Colgate's, and cover it bountifully with precipitated chalk. Or if preferred some *approved* alkaline tooth powder, such as Lyon's, can be used. Rinse the mouth thoroughly and then brush the teeth carefully, thoroughly, and *properly*.

After luncheon, when possible, floss silk should be used, and the mouth most thoroughly rinsed with a wash—otherwise with clear water.

After dinner repeat the above.

Just before retiring the teeth should be again thoroughly and correctly brushed and flossed or preferably carefully taped as in the morning and the mouth thoroughly rinsed with an alkaline wash.

Nothing short of the above constitutes good care of the teeth, when the mouth is in a healthy condition.

If the gums have a tendency to bleed, or there is extreme sensitiveness of the teeth, other treatment may be necessary, which should be directed by the dentist.

Tooth picks should not be used, but if insisted upon, only fine quills.

Wooden picks should never be used.—C. EDMUND KELLS.

### LIME WATER

Lime water is one of the best of mouth washes. Should be made at home and used *profusely*.

Place about a cup full of unslacked lime in a half-gallon bottle, fill with water, cork well and shake several times during the day.

Upon the following morning, pour most of the water, which contains the washings of the lime, and throw away.

Fill again with water, and shake well. When settled, decant carefully into smaller bottles, to be kept upon the toilet stands for daily use. Then fill the jar with water again, and shake well, and you have another jar full of lime water. Used in this manner this amount of lime will furnish lime water for six months or more, if the jar is kept well corked.

For use, add a sufficient quantity (say a table spoon or two) to a half-glass of water so that it can be slightly tasted, and rinse the mouth most thoroughly. If lime water is used too strong it will affect the mouth unpleasantly. Keep the bottles well corked.—C. EDMUND KELLS.

### CARE OF CHILDREN'S TEETH

The proper care of children's teeth is of the utmost importance, and while it is a fact that the temporary teeth will be lost, it is nevertheless true that their neglect may bring about more serious results than equal neglect of the permanent teeth.

As soon as the first tooth is well erupted, the use of the mouth rag should be supplemented by that of a soft brush, and with this and clear water the teeth should be carefully brushed every day.

Immediately upon their full eruption, the surfaces which are in contact with each other should be polished daily by means of suitable floss silk.

When the child has reached an age at which it will not swallow everything that is put into its mouth, precipitated chalk should be used upon the brush once a day.

The mouth should be well rinsed with dilute lime water, upon rising, after meals and just before going to bed.

Later on, cleanse the teeth as follows: Rinse the mouth well. Place a *little* of a good dental cream upon the *dry brush,* and cover the brush bountifully with precipitated chalk and brush as taught.

If the teeth were perfectly formed and the occlusion correct, this should keep them in perfect condition, provided always that the proper diet has been insisted upon and the child taught to properly masticate its food.

Little children should not be allowed to eat candy or other sweets. If there is any agent which will ruin infants' teeth more quickly and more seriously than condensed milk, it is not known to the writer.

Unless there is evidence of such necessity, the child need not be taken to the dentist until it is two years and a half old. At that age the teeth should be carefully examined by him, and again every three or four months.

However, if at any time dark stains accumulate upon the teeth near the margin of the gums, they should be polished off. It is absolutely essential that all surfaces be kept clean and bright.

The decay of these temporary teeth insures the child untold discomfort and pain, and usually interferes with the proper eruption of the second set. Owing to their nature it is most essential that all cavities should be filled in their incipiency.

The extraction of any temporary teeth before the period for the eruption of their permament successors usually interferes with the proper eruption of the second set.

The first teeth of the permanent set to appear are the first molars, which should erupt at about six years of age and *before any of the first set have been lost.* These teeth should be kept under a watchful eye, as they are very prone to decay.

If at this age the arches have not grown, and all the front teeth so separated that one or two thicknesses of blotting paper cannot be put between them, the second set is sure to be crowded and irregular. In such cases, possibly the arches should be expanded and the necessary space made to accommodate the larger teeth of the second set. The family dentist should always be first consulted regarding this condition.

While some children may contract unfortunate habits, notwithstanding the most strenuous efforts being made to prevent their doing so, the permitting of a child to suck its thumb or fingers, or the giving it a ''pacifer,'' is simply criminal, as most serious results must follow.

The harmfulness of mouth breathing should be recognized and the necessary steps taken to cure it.

During the eruption of the second teeth, they should be given special care, and should be examined at least twice a year, and cleaned and polished as often as necessary. Cavities should be filled in their incipiency.

If the family dentist were charged with the duty of sending for the child at stated intervals, the chances of neglect upon the part of the parent would be minimized.

To brush the teeth carefully, thoroughly and properly is quite a trick and cannot be well taught by the absent method. The family dentist should personally explain this to the child and give advice as to the style of brush to use.

Eternal vigilance is the price of good teeth in the child as well as the adult.—C. Edmund Kells.

No one who has spent his life in such a splendid profession as that of dentistry can fail to note with alarm the adoption by the profession of some fad which all of his experience convinces him is bad for the patient. Forty years and more of careful consideration and experience in prophylaxis and dentifrices—forty years and more of clinical evidence—convince me that the use of acid paste, powder, and wash must be detrimental to the teeth, and their use today is largely a fad, mostly among the thoughtless.

Undoubtedly the larger part of the dental profession, and the public as well are absolutely "at sea" upon the question of dentifrices, and as the subject never has received the attention it deserves at the hands of the profession the time has come for this association to take hold of the subject and give it the prominence it deserves. What we desire to be informed upon by some real authority so that we can speak to our patients with confidence upon the subject is:

1. Can a dentifrice, be it powder, or liquid, destroy the harmful germs to be found in the mouth and not injure the teeth themselves?

2. Can any dentifrice do more than cleanse the teeth and mouth?

3. In view of the fact that a normal mouth is either neutral or slightly alkaline, and that acids injure the enamel, should or should not a suitable dentifrice be either neutral or slightly alkaline and thus in harmony with nature, rather than acid, which must necessarily act as a counter-irritant?

4. Should not the dental colleges have a separate chair for the teaching of prophylaxis and the study of dentifrices?

5. Should not manufacturers be discouraged from using misleading advertisements?

This is what we dentists should know. This is what our patients should know, and the only solution of the problem lies in the hands of the National Dental Association, which should appoint a Commission to investigate the subject and work out some suitable plan for its solution.

DISCUSSION BY DRS. G. W. DITTMAR AND R. W. BUNTING

Dr. Kells has presented a forceful and convincing argument in favor of alkaline dentifrices and I cannot disagree with him. There is, however, much confusion and difference of opinion on this subject and after having heard this excellent paper there may still be some who are of the opinion that Dr. Pickerill is right, or that the arguments of the manufacturers of dentifrices containing acid are right. To me Dr. Kells' findings are nothing more than good common sense, though they may not prove that his conclusions are correct. The care of the mouth and especially the teeth is a subject that has received comparatively little attention from a preventive or prophylactic standpoint. It is true that pathological conditions have been studied and the science and art of restoring lost teeth and lost tooth structure has developed almost to perfection, but the science underlying the prevention of most dental diseases is as yet almost entirely in its infancy. The ravages of dental diseases and oral sepsis can never be successfully combatted until genuine and thoroughly scientific means are formulated to prevent these diseases. Dr. Kells is emphatically right when he says that prophylaxis is the keystone upon which the arch of modern dentistry should depend. Preventive dentistry is the type of dentistry that our profession must study more carefully and finally provide effective means for combating that great menace to health, comfort, and aesthetics—dental caries.

Quoting from my President's Address[3] read before the Illinois State Dental Society last March I said: "We are

[3] Published in the *Illinois State Dental Society Proceedings*, 1920.

confronted with the appalling fact that fully 95 per cent of the mouths of all children of school age are diseased.

"We also know that mouth infections lead to serious systemic disorders, both nervous and organic. It is also a fact that clean teeth do not decay, and while most oral diseases are amenable to treatment, it is plainly evident that prevention of dental disease is the goal which our profession should strive to obtain. The archenemy of the teeth is dental caries. If this disease could be prevented the greater part of the inflammatory and suppurative disorders which so often result in alveolar abscesses and chronic suppurative pericementitis or so-called pyorrhea alveolaris, and their train of systemic infections, would never occur. The logical means to prevent this almost universal disease would be to supply the human organism with the proper antibodies, or else a stimulant which would cause the system to produce these antibodies. Whether this be done by a properly prepared and balanced diet supplying the system with the needed vitamines, or by the inoculation of live or dead bacteria, chemicals or what not—it is my earnest hope that some day some remedy can be administered to the child, which will produce an immunity to dental caries.

"It is evident from reliable statistics obtained from many sources that from 3 to 5 per cent of children possess a natural immunity to dental caries.

"We, as dentists, know that in most mouths an immunity gradually establishes itself. We also know that mouths that have become practically immune may become at times violently susceptible again, due, no doubt, to some metabolic disturbances. In turn the disease in these mouths may again gradually lessen and cease. One fact is evident and that is that some element, some substance antidoting dental caries, is possessed from infancy by a very small percentage of humanity, and that in a very large percentage of the remainder this element or substance is gradually developed. *What is this substance? This antibody? And how can it*

*be produced?* Surely some scientifically trained mind will solve this problem and earn the distinction he will so richly deserve, namely be classed with the world's greatest benefactors.''—G. W. Dittmar, *Journal, N. D. A.*, July, 1921.

DENTRIFRICES—DR. R. W. BUNTING

Dr. Kells has touched on the very significant phase of dental practice. It is indeed a unique situation that regarding dentifrices, which are so important a part of our armament, we have so little accurate information and are so dependent on others for production. For the most part our dentifrices are manufactured by various commercial houses over which we have no control. The formulas of their preparations are proprietary and secret and are subject to change without notice. Certain well-established pharmaceutical houses are making every effort to attain an excellence and uniformity of their products, while many small concerns are using very crude and inefficient methods in the production of preparations which are variable and inferior in quality. It is unfortunate that in the selection of the particular dentifrice to be used and recommended to patients the dental profession is guided not by an accurate knowledge of the composition and action of each but rather by the advertising propaganda of the manufacturers or the personality of their representatives.

We all agree with the essayist that this condition of affairs should not be. It certainly is high time that a concerted effort should be made by the profession to standardize the manufacture of dentifrices and to publish more accurate information in regard to them. It is very probable that we shall always be forced to depend on the larger and established pharmaceutical houses for the wholesale manufacture of dentifrices, just as we are dependent on the dental manufacturers for our instruments and other equipment. But we can and should insist that these products be made according to certain standards and that their particular qualities shall be published.

Before we can do this, however, we must first determine what a good dentifrice is, what it should contain, and how it should be compounded. Until we ourselves discover and agree as to the desirable qualities in a dentifrice we cannot with good grace criticize the manufacturer who is making as good a preparation as he knows how to make. This is clearly our problem and when we have worked it out we shall find that the manufacturers for the most part will be very willing to conform their products to well-established facts concerning them.

The most important qualities of dentifrices may be said to be abrasiveness, saponification, reaction, lodgeability, and taste. Considerable variation is seen in the abrasiveness of the common oral preparations. We published (*Jour. Nat. Dental Assoc.,* 1915, p. 247) photomicrographs of nearly thirty of these preparations showing the wide variation in type of gritty substance which they contained. However, the action of these abrasives is very largely modified by the amount of soap and other substances with which they are incorporated. Just recently we have completed a machine designed for accurately testing the abrasive qualities of powders and pastes. The tests are made by applying the various dentifrices to tooth enamel with a brush, 200,000 strokes being given under fairly uniform conditions. From the tests so far made it appears that there are three grades of preparations, namely, those that are markedly abrasive, those which are moderate abrasives, and those which show little or no abrasive action. Herewith is a list and we feel fairly confident in regard to the particular class to which the articles belong:

| *Abrasive* | *Medium* | *Bland* |
|---|---|---|
| Carmi-pyo | Lyon's powder | S. S. White's paste |
| Euthymol powder | Lyon's paste | Chlorax |
| Pepsodent | Colgate's paste | Kolynos |
| | | Pebeco |
| | | Carmi Lustro |
| | | Prophylactic |
| | | Listerine |

However, we might go out next week and buy a sample of any one of these, and find they have moved over to another class. We have no assurance that the manufacturers from week to week and month to month make them uniform. Some originally very strong abrasives, today are not, possibly because some of the manufacturers are waking up to the situation and modifying their product to meet the present condition. It is our intention to undertake a large series of tests with this machine during the coming year by which not only the manufactured products will be checked but also the action of each ingredient of which they are composed. In regard to the reaction of dentifrices we find ourselves in accord with Dr. Kells when he says that clinical practice does not support the universal use of acid preparations. There are certain types of cases in which the saliva is thick and mucinous and the mouth is habitually unclean. In them acid washes and dentifrices often materially assist in cleansing the mouth and maintaining oral hygiene. But in those cases in which the saliva is thin and the teeth are naturally self-cleansing the use of acid mouth preparations will frequently result in hyperacidity and an acceleration of dental caries.

Nor is it certain that strongly alkaline dentifrices should be universally adopted. Following the use of lime water the mucous membranes are frequently irritated, and to many patients its use is decidedly distasteful. Except where a decided acid or alkaline is clearly indicated it seems more reasonable that mouth preparations be either neutral or slightly alkaline.

This is a biological problem with which we are confronted. Since various mouths differ as to their physical condition and physiological function, so, then, should the aids which we prescribe for their prophylaxis vary according to the needs of the case. We cannot, therefore, hope to arrive at any one universal formula for a dentifrice, but rather should certain types of formulas be presented from which preparations of standard abrasiveness, saponification, acidity or alkalinity, etc., may be compounded. In this manner may we select and obtain a proper dentifrice for each individual case.—*Journal, N. D. A.*, July, 1921.

